# Human umbilical cord blood-derived mononuclear cell transplantation: case series of 30 subjects with Hereditary Ataxia

**DOI:** 10.1186/1479-5876-9-65

**Published:** 2011-05-16

**Authors:** Wan-Zhang Yang, Yun Zhang, Fang Wu, Min Zhang, SC Cho, Chun-Zhen Li, Shao-Hui Li, Guo-Jian Shu, You-Xiang Sheng, Ning Zhao, Ying Tang, Shu Jiang, Shan Jiang, Matthew Gandjian, Thomas E Ichim, Xiang Hu

**Affiliations:** 1 Department of Rehabilitation Medicine, Nanshan Affiliated Hospital of Guangdong Medical College, Shenzhen, China; 2 Shenzhen Beike Cell Engineering Research Institution, Shenzhen, China; 3 Department of Neurology and Neurosurgery, Stanford University, Stanford, CA, USA; 4 Medistem Inc, San Diego, CA, USA

## Abstract

**Background:**

The differential diagnosis for hereditary ataxia encompasses a variety of diseases characterized by both autosomal dominant and recessive inheritance. There are no curative treatments available for these neurodegenerative conditions. This open label treatment study used human umbilical cord blood-derived mononuclear cells (CBMC) combined with rehabilitation training as potential disease modulators.

**Methods:**

30 patients suffering from hereditary ataxia were treated with CBMCs administered systemically by intravenous infusion and intrathecally by either cervical or lumbar puncture. Primary endpoint measures were the Berg Balance Scale (BBS), serum markers of immunoglobulin and T-cell subsets, measured at baseline and pre-determined times post-treatment.

**Results:**

A reduction of pathological symptoms and signs was shown following treatment. The BBS scores, IgG, IgA, total T cells and CD3+CD4 T cells all improved significantly compared to pre-treatment values (*P *< 0.01~0.001). There were no adverse events.

**Conclusion:**

The combination of CBMC infusion and rehabilitation training may be a safe and effective treatment for ataxia, which dramatically improves patients' functional symptoms. These data support expanded double blind, placebo-controlled studies for these treatment modalities.

## Background

Hereditary ataxias are a heterogeneous group of neurodegenerative disorders, characterized by degenerative atrophy of the cerebellum, brain stem and/or spinal cord. The primary sequelae are clinical manifestations of dysarthria, dyscoordination of limbs, instability of gait, and eventual loss of posture [1-3]. Spinocerebellar ataxia (SCA) and Friedreich's ataxia (FRDA) are the most common forms of hereditary ataxia. Genetic anticipation usually occurs in familial patients, with symptoms and signs getting more severe with each successive generation [2,3]. The disease is characterized by progressively disabling clinical manifestations. Patients show symptoms of gait instability or dysarthria and may begin to fall without warning. Gradually they present progressive limitations in their activities, lose the ability to walk, become bedridden and fully dependent, and most commonly succumb to pulmonary infection as the cause of death [2,4].

To date, no effective routine therapy is currently available for hereditary ataxia [5-7]. Stem cell therapies were recently studied as an option to treat neurodegenerative disorders as it may provide neuroprotection and possibly promote regeneration [8-13]. In addition, studies on animal models [14,15] and humans [16,17] reported the therapeutic safety and efficacy of stem cell transplantation in cerebellar ataxia. Human umbilical cord blood (hUCB) proved to be a rich source of pluripotent stem cells for clinical application in neurodegenerative diseases [18,19]. The mononuclear cells derived from hUCB are mainly comprised of a heterogenous population of hematopoietic and mesenchymal stem cells, endothelial progenitor cells and immature immunological cells [16,20]. In this study, CBMC transplantation was examined as a potential therapy for hereditary ataxia. Thirty sequential patients with hereditary ataxias were treated with non-matched, allogeneic CBMCs. Treatment included both intravenous and intrathecal infusion of CBMCs, combined with proprioceptive neuromuscular facilitation. Our results indicate this combined treatment improved ataxia patients' functionality and quality of life.

## Methods

### Patient characteristics

Thirty patients with hereditary ataxia were recruited between January 2006 - May 2007 from the Nanshan Affiliated Hospital of Guangdong Medical College. Twenty five subjects had confirmed SCA (Type 1: 1 case, Type 2: 8 cases, Type 3: 5 cases, Type 6: 4 cases, unidentified genotype: 7 cases) and 5 cases of FRDA. The mean age was 43.14 ± 12.77 (range 19 to 71 years). The male-female gender ratio was 18:12. On average, patients had ataxias for 10.74 ± 5.89 years. The longest disease duration at the time of treatment was 26 years. Patients treated came from Australia, Britain, Canada, China, Chile, Italy, South Africa and U.S.A. There were no significant demographic or baseline co-morbidity differences in the 30 subject cohort.

The brain and cord MRI (Symphony 1.5T, Siemens, Germany) confirmed atrophy in the cerebellar hemispheres combined with atrophies at different levels in the brainstem and the cervical and thoracic segments of the spinal cord, but there were no signs of organic changes to the brain parenchyma. As per protocol, the pre- and post-treatment study tested for complete blood counts, routine urine tests, liver function, renal function, electrolytes, sero-enzymology, blood glucose, blood lipids, cellular and humoral immunity, routine cerebro-spinal fluid (CSF) and biochemical markers (biochemistry analyzer, Beckman, US and *Epics-XL *flow cytometer, Beckman, US).

### Clinical treatment

All subjects were hospitalized while receiving CBMC transplantations. The CBMCs were provided by *Shenzhen Beike Biotechnology Co., Ltd*. after hUCB collection and mononuclear cell extraction, cultivation and harvest [16]. Approximately 1-3 × 10^7 ^CBMCs were transfused per injection. Patients received both intrathecal and intravenous injections. The protocol, patient consent, and safety measures were approved by the local institutional review board of the Nanshan Affiliated Hospital of Guangdong Medical College under the auspices of the National Ministry of Health. Patients were explained the experimental nature of the procedure and informed consent was obtained from all patients before initiation of treatment. CBMCs were administered by intravenous infusion combined with intrathecal injection by either cervical or lumbar puncture. Each patient received cell transplantation four to six times - depending on the patient's condition, within an interval of five to seven days. Two ml of CSF was removed and replaced by 2 ml of cell suspension during the intrathecal injection. In terms of intravenous infusion, 30 ml of cell suspension was given through an intravenous catheter over 15-20 minutes. During stem cell treatment, rehabilitation cycles of balance training (proprioceptive neuromuscular facilitation) were given twice daily for four to six weeks, each cycle lasting 30 minutes. The major techniques employed in this training were: (1) visual compensation: the aim was to improve the proprioceptor sensitivity with the help of visual compensation; (2) using balance boards: the states tested were from static to moving; the support tope was from stable to unstable; eyes were from open to closed. A phased and sequenced manner was chosen based on the result of balance evaluation. The basis evaluation of treatment efficacy was executed with the Berg Balance Scale (BBS), which consists of 14 items assessing the ability to stand up and to maintain standing position despite internally produced perturbations [21]. Each item is scored from 0 (unable) to 4 (safely done) with a maximum total score of 56 [21].

### Criterion of therapeutic effect

There were no published criteria to measure therapeutic efficacy in the treatment of ataxia. We applied an accepted statistical methodology of 50% or greater improvement from baseline in BBS score. In order to quantitate the response further, >50% was deemed to be markedly effective; 5%-49% was classified as effective, while <5% improvement was deemed to be ineffective.

### Statistics

Testing was standardized for each sample or examination. Data were presented as means ± standard deviations (). Change in each outcome variables between pre- and post-treatment was assessed using paired T-test. Bonferroni adjustment was made for multiple comparisons within each kind of outcome variables. An outcome variable was considered to be significant if p < 0.05/m, where m = number of comparisons made for each kind of outcomes. All statistical analyses were done using SPSS 13.0 statistical package. All statistical tests were two-sided and a p-value < 0.05 was considered statistically significant.

## Results

Administration of CBMCs via intrathecal and intravenous routes was well tolerated during the clinical treatment course. With treatment, 13/30 BBS score improved by >50% and 17/30 showed improvement between 5% ~ 49%. The highest increase was 87.5% while the lowest one was 18.8%. All showed marked functional effects. The efficacy rate of balancing from these samples was 100% (Table 1). The BBS score improvement was significantly elevated after treatment (Table 2, P < 0.001).

**Table 1:** Efficacy Rate

BBS Score	Improved >50%	Improved 5~49%	Improved <5%	Total
Patient # (n)	13	17	0	30
Efficacy Rate	43.3%	56.7%	0%	100%

**Table 2:** BBS score()

Item	Patient # (n)	Pre-treatment	Post-treatment	P value
BBS Score	30	35.62 ± 11.25	45.25 ± 9.33	< 0.001

Of the immune parameters, there was significant reduction in IgG 9.76 ± 3.079 vs 8.09 ± 2.357 and IgA 2.12 ± 0.808 vs 1.92 ± 0.760. The C3, C4 and IgM measures were not significantly altered (Table 3, P < 0.05/5 = 0.01). Total T cells 78.29 ± 8.011 vs 74.85 ± 8.588 and CD3+CD4 T cells 49.07 ± 8.531 vs 44.93 ± 9.642 were significantly decreased after the treatment (Table 4, P < 0.05/4 = 0.0125).

**Table 3:** Immunoglobulin ()

Item (Unit)	Patient # (n)	Pre-treatment	Post-treatment	P value
C3 (mg/l)	30	1.18 ± 0.247	1.19 ± 0.221	0.921
C4 (mg/l)	30	0.26 ± 0.073	0.25 ± 0.081	0.415
IgG (g/l)	30	9.76 ± 3.079	8.09 ± 2.357	<0.001
IgA (g/l)	30	2.12 ± 0.808	1.92 ± 0.760	0.001
IgM (g/l)	30	1.03 ± 0.792	1.05 ± 0.711	0.677

**Table 4:** T-cell subsets()

Item	Patient # (n)	Pre-treatment	Post-treatment	P value
Total T Cells (%)	30	78.29 ± 8.011	74.85 ± 8.588	0.002
CD3+CD4 (%)	30	49.07 ± 8.531	44.93 ± 9.642	<0.001
CD3+CD8 (%)	30	23.81 ± 7.737	24.71 ± 7.737	0.954
Th/Tc (%)	30	2.29 ± 0.942	2.02 ± 0.815	0.147

## Discussion

The frequency of exact diagnosis and confirmation of hereditary ataxias has risen in tandem with advances in genetic testing that define the different types and the locus of genotype variation, abnormalities within chromosomes and proteins. Mutational analysis can correlate with apoptosis, necrosis or degeneration of neurons in the cerebellum, brain stem, or spinal cord. The rate and quantity of atrophy and degeneration of neurons differ with the various types of hereditary ataxia and patients' ages. The pathological neuronal loss results in loss of cerebellospinal tracts and functional disorders. The physical manifestations translating to functional disability include unsteadiness in walking, wide-based steps, inability to heel walk, unsteadiness in standing or sitting, dependence on a walking frame, walking aid or wheel-chair, dysarthria and dysphagia. Despite genotypic variability, the phenotypic symptoms among patients are mostly similar, only differing in ages of onset or rates of progression. According to the iconography records of the FRDA subjects in this study, the onset of cerebellar atrophy is approximately five years before symptoms appear and the progression is in direct proportion to the atrophy ratio of cerebellum and spinal cord. Patients eventually develop severe functional impairment of swallowing, loss of locomotor capacity and even death due to respiratory muscle paralysis or pulmonary infection.

Prior studies attempt to treat neurodegenerative diseases with human embryonic olfactory ensheathing cell [22] and neural stem cell [17,23] transplantation. However, there are no publications documenting systematic study of hereditary ataxia treatment with CBMCs. Based on our clinical experience, the short-term effect of CBMC transplantation combined with rehabilitation training on equilibrium function treating hereditary ataxia was significant. After receiving one treatment course, the patients were evaluated by physicians and therapists using BBS, a validated functional scale that measures the ability to walk, balance while standing and other activities of daily living for ataxia patients [21].

The average duration of symptoms of the subjects enrolled was over 10 years, and therefore, most received equilibrium function training without significant improvements prior to CBMC treatment. One of the patients with SCA6 who needed complete support while walking and had abnormal Romberg sign (+), heel-knee-tibia test (+) and heel test (+) at baseline, subjectively felt marked improvements immediately after the CBMC transplantation and could objectively walk without support. He also finished the heel test after three CBMC transplantations. In addition, this subject's condition remained stable three years after the treatment according to the follow up examinations.

One family from Saskatchewan, Canada had 32 individuals with confirmed SCA2 from 80 tested family members spanning four generations. Sixteen members of the family had already expired directly from the disease or complications stemming from it. Six male siblings or children from this family participated in the trial. The symptoms in the third generation were relatively mild and all were able to move with support. However, in the fourth generation, symptoms started by age 16 years old. Moreover, all signs and symptoms continued to progress. By age 19, when one fourth generation family member participated, he had already lost his ability to walk. After one course of treatment, his BBS score rose from 26/56 to 43/56. Unfortunately, because of geographical distance, it was impossible to provide long-term follow-up details on all patients who received the treatment.

The interval between baseline and post-treatment of serum IgG, IgA, IgM, C3, C4 and T cell subsets tests, as per protocol, was about a month. IgG, IgA, total T cells and CD3+CD4 T cells decreased significantly after treatment (*P *< 0.01). Although there are numerous proportioned mechanisms of action, one possibility is that CBMCs exercise broad inhibitory action on cellular and humoral immunity. One limitation of the study was that some patients received treatment with CBMC for 20 days in total, whereas others received up to 42 days in total. There were no significant differences in immunological profiles or clinical responses between the 20 to 42 day treatment groups, however this is a question that may be addressed in future studies.

Cord blood derived cells are being investigated in a myriad of preclinical disease models [18,19,24,25]. The safety of CBMC transplantation has been investigated in several human clinical trials with neurodegenerative conditions and has not revealed any severe adverse events, immune reactivity or Graft-versus-host-disease [16,26,27]. The potential concern regarding GVHD induced by allogeneic cord blood administered in absence of immune suppression is mitigated by the fact that hundreds of administrations of allogeneic lymphocytes have been performed in women with recurrent spontaneous abortions as a method of immune modulation, without GVHD being observed [24]. Mechanism studies suggest that multi-potent cells in the heterogeneous CBMC population may not only differentiate into osteoblasts, chondroblasts, adipocytes and neurons and astrocytes to act as a cell replacement source, but also produce antioxidants, several neurotrophic and angiogenic factors and modulate immune and inflammatory reaction [19,28,29]. Intravenously administered CBMCs enter brain, survive, migrate, improve functional recovery and reduce infarct volume in the middle cerebral artery occlusion rat stroke model through the action of anti-inflammatory, neuroprotection and neovascularization [30,31]. Cord blood stem cells infusion into the systemic circulation of G93A mice, an amyotrophic lateral sclerosis (ALS) model, delayed disease progression for 2-3 weeks and increased lifespan of diseased mice by providing cell replacement and protection of motor neurons [32]. Transplantation of hUCB cells into the spinal cord injury (SCI) rats most likely inhibits the apoptotic cascade which is followed by axonal remyelination, regeneration of the damaged neural tissues, potential restoration of blood flow to the damaged area by neovascularization, and modulation of the immune/inflammatory response to the injury [33,34]. Accordingly, these multiple restorative and protective effects from CBMC grafts may act in harmony to exert therapeutic benefits for hereditary ataxias, but the exact mechanism of action still remains unconfirmed.

## Conclusion

This open label single dose treatment case series using CBMC transplantation in 30 subjects with hereditary ataxia demonstrated statistically significant endpoints of functional and surrogate immune marker changes from baseline. In addition to the early effect seen in some subjects, the measured symptomatic improvements persisted throughout the period of the study, as noted with the follow-up data from a subset of subjects. These data suggest a potential treatment using CBMC transplantation for hereditary ataxia and possibly other neurodegenerative conditions involving the spinal cord or cerebellum. Based on the current data, further double-blind placebo controlled studies are warranted to validate the efficacy, safety and long-term effects.

## Competing interests

XH is a shareholder of Beike Biotechnology. No other authors declare any competing interests.

## Authors' contributions

WY conceived of the study, participated in its design and coordination, carried out the clinical treatment and performed the statistical analysis. YZ analyzed and interpreted data and drafted the manuscript. MZ, FW, CL, SL, GS, YS, NZ, YT, Shan-J carried out the clinical treatment and collected data. SC, Shu-J, MG, TI analyzed and interpreted data and helped to draft the manuscript. XH conceived of the study, participated in its design and coordination and helped to draft the manuscript. All authors read and approved the final manuscript.

## References

[B1] Wang WZ Luo ZM Neurology Beijing: The People's Medical Publishing House 2004 5 286 29021592009

[B2] Furtado S Das Sand Suchowersky O A review of the inherited ataxias: recent advances in genetic, clinical and neuropathologic aspects Parkinsonism and Related Disorders 1998 4 161 169 10.1016/S1353-8020(98)00030-3 18591106

[B3] Schmitz-Hübsch T Klockgether T An update on inherited ataxias Current Neurology and Neuroscience Reports 2008 8 310 319 10.1007/s11910-008-0048-4 18590615

[B4] Fogel BL Perlman S Clinical features and molecular genetics of autosomal recessive cerebellar ataxias Lancet Neurol 2007 6 245 257 10.1016/S1474-4422(07)70054-6 17303531

[B5] Brusse E Maat-Kievit JA Swieten JC Diagnosis and management of early- and late-onset cerebellar ataxia Clin Genet 2007 71 12 24 10.1111/j.1399-0004.2006.00722.x 17204042

[B6] Mancuso M Orsucci D Choub A Siciliano G Current and emerging treatment options in the management of Friedreich ataxia Neuropsychiatric Disease and Treatment 2010 6 491 499 10.2147/NDT.S6916 20856912 PMC2938298

[B7] Santos R Lefevre S Sliwa D Seguin A Friedreich Ataxia: Molecular Mechanisms, Redox Considerations, and Therapeutic Opportunities Antioxid Redox Signal 2010 13 651 690 10.1089/ars.2009.3015 20156111 PMC2924788

[B8] Locatelli F Bersano A Ballabio E Lanfranconi S Papadimitriou D Strazzer S Bresolin N Comi GP Corti S Stem cell therapy in stroke Cell Mol Life Sci 2009 66 757 772 10.1007/s00018-008-8346-1 18989624 PMC11131442

[B9] Liang J Zhang H Hua B Wang H Wang J Han Z Sun LY Allogeneic mesenchymal stem cells transplantation in treatment of multiple sclerosis Multiple Sclerosis 2009 15 644 646 10.1177/1352458509104590 19389752

[B10] Martinez HR Gonzalez-Garza MT Moreno-Cuevas JE Caro E Gutierrez-Jimenez E Segura JJ Stem-cell transplantation into the frontal motor cortex in amyotrophic lateral sclerosis patients Cytotherapy 2009 11 1 26 34 10.1080/14653240802644651 19191058

[B11] Roberts TJ Price J Williams SC Modo M Preservation of striatal tissue and behavioral function after neural stem cell transplantation in a rat model of Huntington's disease Neuroscience 2006 139 1187 1199 10.1016/j.neuroscience.2006.01.025 16517087

[B12] Redmond DE Bjugstad KB Teng YD Ourednik V Ourednik J Wakeman DR Parsons XH Gonzalez R Blanchard BC Kim SU Gu Z Lipton SA Markakis EA Roth RH Elsworth JD Sladek JR Sidman RL Snyder EY Behavioral improvement in a primate Parkinson's model is associated with multiple homeostatic effects of human neural stem cells Proc Natl Acad Sci 2007 104 12175 12180 10.1073/pnas.0704091104 17586681 PMC1896134

[B13] Corti S Nizzardo M Nardini M Donadoni C Salani S Ronchi D Saladino F Bordoni A Fortunato F Del BR Papadimitriou D Locatelli F Menozzi G Strazzer S Bresolin N Comi GP Neural stem cell transplantation can ameliorate the phenotype of a mouse model of spinal muscular atrophy J Clin Invest 2008 118 3316 3330 10.1172/JCI35432 18769634 PMC2525699

[B14] Jones J Jaramillo-Merchán J Bueno C Pastor D Viso-León M Martínez S Mesenchymal stem cells rescue Purkinje cells and improve motor functions in a mouse model of cerebellar ataxia Neurobiol Dis 2010 40 2 415 423 10.1016/j.nbd.2010.07.001 20638477

[B15] Chintawar S Hourez R Ravella A Gall D Orduz D Rai M Bishop DP Geuna S Schiffmann SN Pandolfo M Grafting neural precursor cells promotes functional recovery in a SCA1 mouse model J Neurosci 2009 29 42 13126 13135 10.1523/JNEUROSCI.0647-09.2009 19846700 PMC6665209

[B16] Yang WZ Zhang Y Wu F Min WP Minev B Zhang M Luo XL Ramos F Ichim TE Riordan NH Hu X Safety evaluation of allogeneic umbilical cord blood mononuclear cell therapy for degenerative conditions Journal of Translational Medicine 2010 8 75 80 10.1186/1479-5876-8-75 20682053 PMC2922090

[B17] Zhang JW Guo SG Wang CJ Sun T Wang TH Treatment of cerebellar ataxia by neural stem cell transplantation Journal of Medical Forum 2010 31 18 19 20

[B18] Garbuzova-Davis S Sanberg CD Kuzmin-Nichols N Willing AE Gemma C Bickford PC Miller C Rossi R Sanberg PR Human umbilical cord blood treatment in a mouse model of ALS: optimization of cell dose PLoS One 2008 3 6 e2494 10.1371/journal.pone.0002494 18575617 PMC2429976

[B19] Park DH Borlongan CV Willing AE Eve DJ Cruz LE Sanberg CD Chung YG Sanberg PR Human umbilical cord blood cell grafts for brain ischemia Cell Transplant 2009 18 9 985 998 10.3727/096368909X471279 19523333

[B20] Javed MJ Mead LE Prater D Bessler WK Foster D Case J Goebel WS Yoder MC Haneline LS Ingram DA Endothelial Colony Forming Cells and Mesenchymal Stem Cells are Enriched at Different Gestational Ages in Human Umbilical Cord Blood Pediatr Res 2008 64 68 73 10.1203/PDR.0b013e31817445e9 18360305

[B21] Yelnik A Bonan I Clinical tools for assessing balance disorders Neurophysiol Clin 2008 38 6 439 445 10.1016/j.neucli.2008.09.008 19026963

[B22] Chen L Jiang Z Huang HY Zhang F Liu YC Xi HT Wang HM Ren YS Zhou CM Preliminary Result of Olfactory Ensheathing Cell Transplantation in Intractable Neuropathic Pain Following Spinal Cord Injury: 17 Cases Report Chinese Journal of Rehabilitation Theory and Practice 2010 16 2 146 148

[B23] Zhang RY Zheng YR Hu SS Cheng HB An YH Clinical analysis of neural stem cells for treatment of sequela in 50 stroke patients Chinese Journal of Clinical Rehabilitation 2006 20 9 138 139

[B24] Riordan NH Chan K Marleau AM Ichim TE Cord blood in regenerative medicine: do we need immune suppression? J Transl Med 2007 5 8 10.1186/1479-5876-5-8 17261200 PMC1796850

[B25] Yang WZ Wu F Zhang M Yang JY Liang HM Sheng YX Hu X Zhao N Yang ZG Tang Y Cord Blood-derived Neural Stem Cell Transplantation in Nervous System Diseases Chinese Journal of Integrative Medicine on Cardio-/Cerebrovascular Disease 2009 7 3 287 290

[B26] Yang WZ Yang JY Liang HM Zhang M Wu F Sheng FX Yang ZG Tang Y Zhao N Hu X Jiang S Guo SQ Efficacy and Safety of Cord Blood Source Neural Stem Cell for Amyotrophic Lateral Sclerosis Chinese Journal of Integrative Medicine on Cardio-/Cerebrovascular Disease 2007 5 10 911 913

[B27] Zhang M Yang WZ Sheng YX Wu F Liang HM Zhao N Hu X Jiang S Guo SQ Clinical Efficacy and Safety of Transplantation of Umbilical Cord Blood Source Neural Stem Cells for Multiple Sclerosis Practical Preventive Medicine 2008 15 4 1186 1188

[B28] Newcomb JD Sanberg PR Klasko SK Willing AE Umbilical cord blood research: current and future perspectives Cell Transplant 2007 16 2 151 158 17474296 PMC2720821

[B29] Arien-Zakay H Lecht S Bercu MM Tabakman R Kohen R Galski H Nagler A Lazarovici P Neuroprotection by cord blood neural progenitors involves antioxidants, neurotrophic and angiogenic factors Exp Neurol 2009 216 1 83 94 10.1016/j.expneurol.2008.11.006 19070617

[B30] Vendrame M Cassady J Newcomb J Butler T Pennypacker KR Zigova T Sanberg CD Sanberg PR Willing AE Infusion of Human Umbilical Cord Blood Cells in a Rat Model of Stroke Dose-Dependently Rescues Behavioral Deficits and Reduces Infarct Volume Stroke 2004 35 2390 2395 10.1161/01.STR.0000141681.06735.9b 15322304

[B31] Chen J Sanberg PR Li Y Wang L Lu M Willing AE Sanchez-Ramos J Chopp M Intravenous Administration of Human Umbilical Cord Blood Reduces Behavioral Deficits After Stroke in Rats Stroke 2001 32 2682 2688 10.1161/hs1101.098367 11692034

[B32] Garbuzova-Davis S Willing AE Zigova T Saporta S Justen EB Lane JC Hudson JE Chen N Davis CD Sanberg PR Intravenous administration of human umbilical cord blood cells in a mouse model of amyotrophic lateral sclerosis: distribution, migration, and differentiation J Hematother Stem Cell Res 2003 12 3 255 270 10.1089/152581603322022990 12857367

[B33] Park DH Lee JH Borlongan CV Sanberg PR Chung YG Cho TH Transplantation of Umbilical Cord Blood Stem Cells for Treating Spinal Cord Injury Stem Cell Rev 2011 7 1 181 194 10.1007/s12015-010-9163-0 20532836

[B34] Dasari VR Spomar DG Gondi CS Sloffer CA Saving KL Gujrati M Rao JS Dinh DH Axonal Remyelination by Cord Blood Stem Cells after Spinal Cord Injury J Neurotrauma 2007 24 2 391 410 10.1089/neu.2006.0142 17376002 PMC1859845

